# Pharmacopuncture for the management of musculoskeletal diseases: A protocol for systematic review

**DOI:** 10.1097/MD.0000000000019082

**Published:** 2020-02-07

**Authors:** Ji Hye Hwang, Jaseung Ku, Jin-Ho Jeong

**Affiliations:** a Department of Acupuncture & Moxibustion Medicine, College of Korean Medicine, Gachon University, Seongnam; b Bogwang Korean Medical Clinic; c Jisung-Kyunghee Korean Medicine Clinic, Seoul, Republic of Korea.

**Keywords:** aqua-acupuncture, herbal acupuncture, musculoskeletal disease, pharmacopuncture, protocol, systematic review

## Abstract

**Background:**

Musculoskeletal disorders are the main reason for people to seek counseling and use of complementary and alternative medicine. Although pharmacopuncture is used to treat various diseases in traditional medicine, it is most often applied to treat musculoskeletal conditions. Here, we will review systematically the clinical evidence for the effectiveness and safety of pharmacopuncture for musculoskeletal diseases.

**Methods:**

A total of 13 databases will be searched for studies uploaded from January 2014 to December 2018 that investigated the treatment of musculoskeletal diseases. These databases are MEDLINE, EMBASE, AMED, Cochrane Library, CINAHL, 4 Korean databases, 2 Chinese database, and 2 Japanese databases. The methodological quality of *randomized controlled trials* will be analyzed using the Cochrane Collaboration tool to assess risk of bias, and the confidence in the cumulative evidence will be assessed using the Grading of Recommendations Assessment, Development and Evaluation (GRADE) instrument.

**Ethics and dissemination:**

This systematic review will be published in a peer-reviewed journal and disseminated electronically and in print. To inform and guide healthcare practices, the review will be updated.

**Registration number:**

CRD42019148795.

## 1 Introduction

Musculoskeletal diseases, especially low back and neck pain, are the main reason for people to seek counseling and use of complementary and alternative medicine.^[1,2]^ According to 2016 National Health Insurance Statistics of Korea, the disease most frequently treated by traditional Korean medicine (TKM) was musculoskeletal-related disease; and about one-third of approximately 16.47 million patients with total musculoskeletal disorders used TKM institutions, and TKM total medical expenses were about 1.4 trillion Korean won.

Pharmacopuncture (or herbal acupuncture or aqua-acupuncture) is a new form of acupuncture method that combines acupuncture with the injection of herbal medicine extracts based on pharmacology and meridian theory, and it can provide the effects of both acupuncture and herbal medicine. Compared with traditional acupuncture, the main advantages of pharmacopuncture are more rapid effects, ease of dosage adjustment, and additional synergistic effects from the acupuncture and herbal medicine extracts.^[3,4]^ The number of clinical trials associated with pharmacopuncture has increased since the 2000s, and the conditions most frequently addressed in past experimental/clinical studies related to pharmacopuncture were musculoskeletal diseases.^[5]^ Currently, numerous kinds of herbal extracts are used in pharmacopuncture treatment. Pharmacopuncture has been applied to treat various diseases, but it has been applied most often on musculoskeletal conditions, the effects of which have been well studied.^[6,7]^

In Korea and China, pharmacopuncture has different origins. It began to be studied in earnest in the 1950s and 1960s, and various changes in techniques occurred in the 1980s. Now it is one of the representative treatment methods of TKM and traditional Chinese medicine (TCM). In China, studies on pharmacopuncture have mainly involved a union of Chinese and Western medicine, while the theory of TCM has tended to be poorly addressed.^[8,9]^ In Korea, although pharmacopuncture is applied extensively, systematic reviews on pharmacopuncture related clinical studies including detailed instructions and side effects are still insufficient.^[10,11]^ In this study, we will review systematically randomized controlled trials (RCTs) to evaluate the effectiveness and safety of pharmacopuncture based on traditional medicine for musculoskeletal diseases.

## 2 Methods

### 2.1 Study registration

The current protocol report complies with the Preferred Reporting Items for Systematic Reviews and Meta-Analyses (PRISMA) protocols.^[12]^ The protocol for this systematic review was registered on PROSPERO, the international prospective register of systematic reviews, under the number CRD42019148795.

### 2.2 Dissemination and ethical approval

This systematic review will be published in a peer-reviewed journal and disseminated electronically and in print. The review will be updated to inform and guide health care practices. As this is a study based on a review of published literature, ethical approval is not required.

### 2.3 Data sources

Databases and search terms will be determined through discussion between all authors before the literature searches are executed. Two authors will perform the electronic literature searches. The following electronic databases will be searched for studies uploaded from January 2014 to December 2018 that investigated the treatment of musculoskeletal diseases: MEDLINE, EMBASE, AMED, Cochrane Library, CINAHL, four Korean databases (KoreaMed, Oriental Medicine Advanced Searching Integrated System (OASIS), the Korean Studies Information Service System (KISS), and Korean Traditional Knowledge Portal (KTCKP)), 2 Chinese databases (China National Knowledge Infrastructure (CNKI) and WanFang Data), and 2 Japanese databases (CiNii and Japanese Institutional Repositories Online (JAIRO)).

### 2.4 Types of studies

Prospective RCTs that evaluate the effectiveness of pharmacopuncture as a treatment for musculoskeletal diseases will be included in this review. Non-randomized trials, literature research, animal or cell studies, and quasi-RCTs (methods of allocating participants to a treatment group that are not actually random) will be excluded. Trials including healthy participants will be excluded. No language restrictions will be imposed.

### 2.5 Types of participants

All RCTs evaluating pharmacopuncture treatment on various musculoskeletal conditions, such as arthritis, HNP of C or L -spine, and sprains, will be considered.

### 2.6 Types of interventions and controls

RCTs that include pharmacopuncture treatment as the sole treatment will be included. Studies that evaluated the combined effects of pharmacopuncture plus other interventions (for example, pharmacopuncture plus acupuncture) will be also considered when the same intervention was carried out in both the pharmacopuncture group and the control group. We will not include RCTs testing injection of bee-venom or conventional medicine because they were not investigated the chemical effects of a herbal medicine.

For control groups, we will consider placebo or sham, no interventions, and other interventions. Placebo or sham interventions can be injections of distilled water or normal saline into the acupuncture points or nonacupuncture points. Other interventions can include acupuncture, herbal/western medicine, cupping, chuna, physiotherapy, and diet therapy, including hot pack, interferential current therapy, transcutaneous electrical nerve stimulation, ultrasound, exercise, and massage.

### 2.7 Outcome measures

#### 2.7.1 Primary outcomes

Pain intensity with relevant confirmed pain measurement scales, such as the visual analogue scale (VAS), verbal rating scale (VRS), and numerical rating scale (NRS), or quality of life will be analyzed.

#### 2.7.2 Additional outcomes

For pharmacopuncture interventions, in addition to adverse events, we will summarize each item in terms of the types and methods of pharmacopuncture, regimen, pharmacopuncture points, extraction methods, types of syringe, and amount, depth, and angle of the injection in accordance with STRICTA recommendations, and the data will be modified into a suitable form for trials of pharmacopuncture.

### 2.8 Data extraction

We will review all searched articles to evaluate their eligibility for inclusion. In the case of uncertainties, authors will be contacted for further information. After the selection of studies, we will extract the following data from the selected articles: author, year of publication, study design, participants (age, gender), diseases or disorders, pharmacopuncture intervention, control intervention, outcome measures, main results, and adverse events.

Two reviewers (JK and JHJ) will conduct the data extraction with a recognized data extraction form agreed on by all reviewers, including author, age, country, year of publication, characteristics of participants, intervention, randomized method, blinding, control treatment, main outcomes, and adverse events. Also they will perform quality assessment using a predefined data extraction form.

### 2.9 Assessment of risk of bias in individual studies

Risk of bias will be assessed using the Cochrane Handbook risk of bias assessment tool version 5.1.0, which takes into account random sequence generation, allocation concealment, blinding of participants and personnel, blinding of outcome assessment, incomplete outcome data, selective reporting, and other sources of bias. The results of the assessments will be presented using the scores of “L” indicating a low risk of bias, “U” indicating an uncertain risk of bias, and “H” indicating a high risk of bias. Any disagreement will be resolved through discussion among all authors; and, if any disagreement regarding selection cannot be resolved through the discussion, an arbiter (JHH) will make the final decision.

### 2.10 Data synthesis

Differences between the intervention and control groups will be assessed. Mean differences (MDs) with 95% confidence intervals (CIs) will be used to measure the effects of treatment for continuous data. We will convert other forms of data into MDs. For outcome variables on different scales, we will use standard MDs with 95% CIs. For dichotomous data, we will present treatment effects as relative risks (RRs) with 95% CIs; other binary data will be converted into RR values.

All statistical analyses will be conducted using Cochrane Collaboration's software program Review Manager version 5.3 (Copenhagen, The Nordic Cochrane Centre, the Cochrane Collaboration, 2014) for Windows. We will contact the corresponding authors of studies with missing information to acquire and verify the data whenever possible. When appropriate, we will pool the data across studies to conduct a meta-analysis using fixed or random effects. We will use GRADEpro software from Cochrane Systematic Reviews to create a Summary of Findings table.

## 3 Discussion

The purpose of this systematic review is to evaluate the effectiveness and safety of pharmacopuncture for musculoskeletal diseases.

In China, pharmacopuncture (aqua acupunture) has been used since the 1950s, and pill-type herbs and all kinds of various medicines that can be injected intramuscularly, from medicinal herbs to Western medicines, have been used as materials in pharmacopuncture. Also, pharmacopuncture has been mainly studied as a union of Chinese and Western medicine, while pharmacopuncture studies involving the theory of TCM have tended to be poorly addressed.^[5]^

In Korea, pharmacopuncture was officially introduced to the TKM community by Sang-Cheon Nam in 1967.^[3–5]^ A previous systematic review on pharmacopuncture dealt with pharmacopuncture-related RCT studies in Korea before 2014 for all diseases, and some of them dealt with musculoskeletal disorders.^[13]^

Therefore, we will review thoroughly research on pharmacopuncture using herbal medicines since 2014, with a focus on musculoskeletal disorders.

Although it can be assumed that pharmacopuncture is a regular part of TKM clinical practice, there are currently few pharmacopuncture-related reports, including clinical practice patterns^[10]^ and side effects^[11]^; and TKM has not yet fully established standardized pharmacopuncture guidelines by disease.^[10]^

A lack of investigations on the type, duration, application, and dosage of pharmacopuncture exacerbates the difficulties in the standardization of pharmacopuncture treatment methods and the development of clinical practice guidelines; and, in addition, a lack of data on clinical applications can be an obstacle to health insurance inclusion, potentially leading to increased health-related costs.^[10]^

This evidence will be useful to patients, practitioners, and health policy makers.

Patients with musculoskeletal diseases will be able to receive appropriate pharmacopuncture treatment, and practitioners will be able to confirm the basis of a decision for treatment.

The study outcomes are also expected to provide basic information for establishing standardized pharmacopuncture guidelines and determining health insurance coverage for pharmacopuncture and standardization of pharmacopuncture.

## Author contributions

JHH and JHJ conceived the study, developed the criteria, JHJ and JK searched the literature, and analyzed the data. JHH wrote the protocol and revised the manuscript. All authors have read and approved the final manuscript.

jihye hwang orcid: 0000-0002-6304-1972.
